# Diet Quality throughout Early Life in Relation to Allergic Sensitization and Atopic Diseases in Childhood

**DOI:** 10.3390/nu9080841

**Published:** 2017-08-05

**Authors:** Anh N. Nguyen, Niels J. Elbert, Suzanne G. M. A. Pasmans, Jessica C. Kiefte-de Jong, Nicolette W. de Jong, Henriëtte A. Moll, Vincent W. V. Jaddoe, Johan C. de Jongste, Oscar H. Franco, Liesbeth Duijts, Trudy Voortman

**Affiliations:** 1 Department of Epidemiology, Erasmus MC, University Medical Center, 3000 CA Rotterdam, The Netherlands; a.n.nguyen@erasmusmc.nl (A.N.N.); j.c.kiefte-dejong@erasmusmc.nl (J.C.K.-d.J.); v.jaddoe@erasmusmc.nl (V.W.V.J.); o.franco@erasmusmc.nl (O.H.F.); 2 The Generation R Study Group, Erasmus MC, University Medical Center, 3000 CA Rotterdam, The Netherlands; n.j.elbert@erasmusmc.nl; 3 Department of Dermatology, Erasmus MC, University Medical Center, 3000 CA Rotterdam, The Netherlands; s.pasmans@erasmusmc.nl; 4 Department of Pediatrics, Erasmus MC, University Medical Center, 3000 CA Rotterdam, The Netherlands; h.a.moll@erasmusmc.nl; 5 Department of Global Public Health, Leiden University College, 3595 DG The Hague, The Netherlands; 6 Department of Internal Medicine, Division of Allergology, Erasmus MC, University Medical Center, 3000 CA Rotterdam, The Netherlands; n.w.dejong@erasmusmc.nl; 7 Department of Pediatrics, Division of Respiratory Medicine and Allergology, Erasmus MC, University Medical Center, 3000 CA Rotterdam, The Netherlands; j.c.dejongste@erasmusmc.nl (J.C.d.J.); l.duijts@erasmusmc.nl (L.D.); 8 Department of Pediatrics, Division of Neonatology, Erasmus MC, University Medical Center, 3000 CA Rotterdam, The Netherlands

**Keywords:** diet quality, allergic sensitization, allergy, eczema, asthma, pregnancy, infants, cohort

## Abstract

Early-life nutrition is an important modifiable determinant in the development of a child’s immune system, and may thereby influence the risk of allergic sensitization and atopic diseases. However, associations between overall dietary patterns and atopic diseases in childhood remain unclear. We examined associations of diet quality in early life with allergic sensitization, self-reported physician-diagnosed inhalant and food allergies, eczema, and asthma among 5225 children participating in a population-based cohort in the Netherlands. Diet was assessed during pregnancy, infancy, and childhood using validated food-frequency questionnaires. We calculated food-based diet quality scores (0–10 or 0–15), reflecting adherence to dietary guidelines. At age 10 years, allergic sensitization was assessed with skin prick tests. Information on physician-diagnosed inhalant and food allergies, eczema, and asthma was obtained with questionnaires. We observed no associations between diet quality during pregnancy and allergic sensitization (odds ratio (OR) = 1.05 per point in the diet score, 95% confidence interval (CI): 0.99, 1.13), allergies (0.96, 95% CI: 0.88, 1.04), eczema (0.99, 95% CI: 0.93, 1.06), or asthma (0.93, 95% CI: 0.85, 1.03) in childhood. Also, diet quality in infancy or childhood were not associated with atopic outcomes in childhood. Our findings do not support our hypothesis that a healthy dietary pattern in early life is associated with a lower risk of allergic sensitization or atopic diseases in childhood.

## 1. Introduction

The prevalence of childhood atopic diseases, such as eczema and food allergy, has increased in the recent decades [1]. These diseases have a substantial impact on the quality of life of those affected [2]. Genetic background is one of the factors associated with atopic diseases, but given the rapid increase in the prevalence, environmental risk factors, including geographic area and lifestyle factors may play a substantial role in the development of allergies and other atopic diseases [3,4,5]. Early-life nutrition is an important modifiable lifestyle factor that influences the development of a child’s immune system [6]. Suboptimal nutrition during pregnancy, infancy, or childhood may interrupt the maturation process of the immune system from fetal life until childhood [6,7], which may increase sensitization and thereby the risk of atopic diseases in childhood. There has been great interest in early-life dietary exposures in relation to atopic diseases, with studies focusing on breastfeeding [8], timing of solid food introduction [9,10], food allergen avoidance [11,12], or intake or blood levels of specific nutrients during pregnancy or in infancy [13,14,15,16]. Although these specific nutritional factors may indeed be relevant for atopic health, these factors may also represent an overall dietary pattern. Individuals do not consume one specific nutrient or food at a time, but a variety of nutrients combined in foods and meals that may interact. Studying overall dietary patterns takes these potential interactions into account [17] and may be more applicable in clinical practice.

A few previous studies examined dietary patterns in relation to atopic diseases. So far, most studies mainly focused on a Mediterranean diet in pregnant women or children in relation to atopic diseases [18,19,20]. However, findings are inconsistent and most of these studies only examined self-reported atopic diseases, such as asthma or allergic rhinitis [18,19,20]. Assessing atopy using skin prick tests may be more sensitive and less affected by measurement error, but only a few studies examined associations between dietary patterns and objectively measured atopy. A study in Spain observed an inverse association between a Mediterranean diet during pregnancy and atopy in childhood [21], whereas other studies did not observe associations of either data-driven dietary patterns [22,23] or predefined dietary patterns (i.e., Mediterranean diet score and Alternate Healthy Eating Index) [23] during pregnancy with atopy in children. A few other studies focused on early childhood dietary patterns in relation to atopic outcomes. For example, a nested case-control study in the United Kingdom found better adherence to a dietary pattern including high intakes of fruits, vegetables, and home-prepared foods in two-year-old children without food allergy than children who did have a diagnosis of food allergy [24]. Recently, a population-based cohort in Singapore reported an inverse association of a dietary pattern high in noodles and seafood at the age of one year with allergic sensitization to house dust mite at the ages of 18 months and five years, but not with self-reported eczema or rhinitis [25]. Finally, results from other population-based cohorts, including previous analyses in our cohort, showed positive associations of a Western dietary pattern with self-reported asthma symptoms in children aged 3–4 years [26] and 8–11 years [27], but not with allergic sensitization measured by skin prick tests [27]. These previous studies examined diet at different points in early life, focused on different types of dietary patterns, and did not adjust for diet at other points in childhood. In addition, previous studies mainly focused on a traditional Mediterranean diet or examined data-driven dietary patterns, which may not represent actual healthy diets and which cannot be extrapolated to other populations because these patterns are population-specific.

Therefore, we aimed to examine the associations between predefined dietary patterns based on Dutch dietary guidelines (i.e., diet quality) during pregnancy, infancy, and childhood with allergic sensitization, inhalant and food allergy, eczema, and asthma in mid-childhood. In addition, we examined whether associations of early-life diet were independent of diet at other time points, including current child diet, and whether associations differed between boys and girls, by maternal history of atopic diseases, and between those who received breastfeeding for at least four months exclusively, partially, or not at all.

## 2. Methods

### 2.1. Study Design and Population

This study was embedded in the Generation R Study, an ongoing population-based prospective cohort from fetal life onward in Rotterdam, the Netherlands [28]. Pregnant women with an expected delivery date between April 2002 and January 2006 were invited to participate. Parents of all participating children provided written informed consent and medical ethical approval was obtained from the medical ethical committee of Erasmus University Medical Center, Rotterdam (MEC 198.782/2001/31, 2001). Further information on the design of the Generation R Study is available elsewhere [28].

In total, parents of 5225 children provided consent, had dietary data for at least one time point (i.e., during pregnancy, infancy, or childhood) and had valid data for at least one of the outcome variables (i.e., sensitization, allergy, eczema, or asthma). Because data on dietary intake at the different time points and the atopic outcomes were not complete for all participants, the population for analysis varied per specific analysis (*n*: between 2519 and 3776) (Supplemental Figures S1–S3).

### 2.2. Dietary Intake during Pregnancy

Dietary intake in early pregnancy (median 13.6 weeks of gestation (interquartile range (IQR) 12.4–16.2)) was assessed using a semi-quantitative food-frequency questionnaire (FFQ). The FFQ included foods that were frequently consumed in the Dutch population and was modified for use in pregnant women. Energy intakes were calculated using data from the Dutch Food Composition Table. The FFQ was validated against three 24-h recalls among 71 pregnant women living in Rotterdam. Intra-class correlation coefficients for macronutrient intakes ranged from 0.5 to 0.7.

We applied a previously developed predefined food-based diet quality score for pregnant women, reflecting adherence to dietary guidelines, as described in detail elsewhere [29]. Briefly, this diet quality score included continuous scores on 15 components: high intake of vegetables, fruit, whole grains, legumes, nuts, dairy, fish, tea; ratio whole grains of total grains, and ratio soft fats (i.e., soft margarines) and oils of total fat; low intake of red meat, sugar-containing beverages, alcohol, salt; and folic acid supplement use in early pregnancy. The maximum score for each component was 1, resulting in an overall sum-score ranging from 0 to 15. A higher score represented a better diet quality [29]. More details on the included components and cut-offs are described in Supplemental Table S1 and elsewhere [29].

### 2.3. Dietary Intake in Infancy

Dietary intake of the children at a median age of 12.9 months (IQR 12.7–14.0) was assessed using a semi-quantitative FFQ, which was developed specifically for Dutch 1-year-old children and filled out by the parents [30,31]. Energy and nutrient intakes of the children were calculated using the Dutch Food Composition Table. The FFQ was validated against three 24-h recalls among 32 children and reasonable to good intra-class correlation coefficients for nutrient intake of 0.4 to 0.7 were found [30,31].

We applied a previously constructed predefined diet quality score for preschool children [30]. As described in detail elsewhere [30], this continuous score reflected adherence to dietary guidelines for preschool children and included ten components, resulting in an overall sum-score ranging from 0 to 10, with a higher score representing a healthier diet [30]. More details on the included components and cut-offs are described in Supplemental Table S2 and elsewhere [30].

### 2.4. Diet Quality in Childhood

Dietary intake in childhood at a median age of 8.1 years (IQR 8.0–8.2) was assessed with a semi-quantitative FFQ, as described in detail elsewhere [32,33]. The FFQ developed for Dutch children in this age group and was filled out by the parents. Energy intakes were calculated using data from the Dutch Food Composition Table. The FFQ was validated for energy intake using the doubly labelled water method (Pearson’s *r* = 0.62) [33].

We applied a previously developed diet quality score for school age children [32], with a similar scoring system as used for the pregnant women and infants. This child diet quality score included ten components, resulting in an overall diet quality sum score ranging from 0 to 10. More details on this diet quality score and the included components are described in Supplemental Table S3 and elsewhere [32].

### 2.5. Allergic Sensitization and Atopic Diseases

Children visited our research center at a median age of 9.7 years (IQR 9.6–9.9). Sensitization to inhalant (including house dust mite, 5-grass mixture, birch, cat, and dog) and food (including peanut, cashew nut, hazelnut, and peach) allergens was assessed by skin prick tests using the scanned area method [34]. Histamine dihydrochloride (10 mg/mL) was used as a positive control in duplicate and a saline solution (NaCl 0.9%) as a negative control. Skin responses were measured 15 min after applying allergens to the skin by measuring the area of the wheal (mm^2^). An area that was ≥40% of the histamine response was considered positive [35]. Children with a positive skin response to any of the allergens were categorized as ‘any allergic sensitization’. We further categorized children into inhalant allergic sensitization and food allergic sensitization. In addition, questionnaires including questions adapted from the International Study of Asthma and Allergies in Childhood core questionnaire [36] were used to obtain information on physician-diagnosed inhalant (‘Was your child ever diagnosed by a physician with an allergy to pollen (hay fever)/house dust mite/cat/dog?’) (no; yes) and food allergies (‘Was your child ever diagnosed by a physician with an allergy to cashew nut/peanut?’) (no; yes). Based on these questions, we dichotomized children into ‘any allergy’ (no; yes). Finally, we further categorized children into ‘sensitization to any allergen and any allergic symptom’ versus ‘no sensitization and no symptoms’. Information on ever eczema and asthma at the age of 10 years was obtained with the same questionnaire (‘Was your child ever diagnosed by a physician with eczema/asthma?’) (no; yes).

## 3. Covariates

At enrollment in the study, maternal height and weight were measured and body mass index (BMI) was calculated (kg/m^2^). Questionnaires were used to obtain information on educational level of the mother (low; high), net household income (<2200 or ≥2200 Euros/month), parity (nulliparous; multiparous), prenatal pet exposure (yes; no), and whether mothers drank alcohol (never; until pregnancy was known; continued drinking occasionally; continued drinking frequently), smoked (never; until pregnancy was known; continued smoking during pregnancy), and used folic acid supplements (no; started in first ten weeks; started periconceptional) during pregnancy. We used questionnaires to obtain information on maternal history of atopic disease, including allergy (hay fever/house dust mite/food), eczema, or asthma. If a mother reported to have any of these outcomes, we categorized her as having a history of atopic disease.

Information on child’s date of birth and sex was obtained from medical records. The child’s ethnic background (Dutch; non-Dutch) was defined based on the country of birth of the parents, which was obtained with questionnaires at enrollment. Information on breastfeeding during the first four months (never; partial; exclusive) was obtained via postnatal questionnaires. Exclusive breastfeeding was defined as receiving breastmilk only for at least four months. Timing of solid food introduction in the first year of life (<3; 3–6; ≥6 months) was obtained from the FFQ administered in infancy. Questionnaires were used to obtain information on day care attendance in the first year of life (≤24 or >24 h/week).

## 4. Statistical Analyses

Non-linearity of associations of diet quality during pregnancy, infancy, or childhood with all atopic outcomes was explored using natural cubic splines (degrees of freedom = 3). As no indications for non-linear associations for the main models were found, all analyses were performed using models assuming linearity. Multivariable logistic regression analyses were used to analyze the associations of either diet quality during pregnancy, infancy, or childhood with allergic sensitization and atopic diseases around the age of 10 years. All associations were analyzed in three models with stepwise adjustment for potential confounders based on previous evidence. The first model was adjusted for child’s ethnic background, sex, age at outcome assessment, and total energy intake. The second model was additionally adjusted for several socioeconomic and lifestyle factors, including maternal BMI at enrollment, maternal educational level, household income, parity, prenatal pet exposure, alcohol intake during pregnancy, smoking during pregnancy, folic acid supplements during pregnancy, and maternal history of atopic disease. In the final model, we examined whether associations of diet quality in pregnancy, infancy, or childhood with allergic sensitization and atopic diseases were independent of diet at the other two time points by additionally adjusting them for each other. Breastfeeding, child’s sex, child’s ethnic background, and maternal history of atopic diseases were separately examined as potential effect modifiers by including interaction terms in the models.

As sensitivity analyses, we repeated our analyses restricted to participants with a Dutch ethnic background only to reduce the risk of residual confounding by ethnicity, since the FFQs were developed for a Dutch population. Also, we repeated our analyses excluding children with any allergic disease in the first year of life for the analyses on infant and child diet quality. In addition, we examined associations of diet quality with the combination of sensitization and allergic symptoms versus no sensitization or symptoms as outcome. Furthermore, we examined whether associations of early-life diet quality with allergic sensitization and atopic diseases were independent of the other outcomes by adjusting associations with atopic diseases for allergic sensitization and vice versa. Finally, to verify that any associations of the overall diet quality scores were not driven by any specific component of the score, we repeated the main analyses excluding one component from the diet score at a time.

To reduce potential bias due to missing values on some of the covariates (ranging from 0 to 30.1%), these variables were multiple imputed (*n* = 10 imputations). Diet quality scores at different time points were treated as either exposure or confounders in the different models. When diet quality was included as a confounder, multiple imputed values of diet quality scores were used and when diet quality was the exposure of interest, the non-imputed variable was used. The results presented are the pooled regression coefficients of the 10 imputed datasets. All statistical analyses were carried out using the statistical software program SPSS statistics version 21.0 (IBM Inc., Armonk, NY, USA).

## 5. Results

### 5.1. Population Characteristics

Characteristics of the study population are presented in Table 1. The majority of the children had a Dutch ethnic background (63.7%), and half of the children were girls (50.8%). Mean (±SD) diet quality score during pregnancy was 7.7 (±1.6) out of theoretical range of 0 to 15, mean diet quality score in infants was 4.3 (±1.4) out of 10, and mean diet quality in 8-year-old children was 4.5 (±1.2) out of 10. None of the participants (either pregnant women or their children) reached the maximum diet quality score. In total, 26.0% of the children were sensitized to one or more allergens, with 25.6% of all children being sensitized to an inhalant allergen and 5.7% to a food allergen. A physician diagnosed allergy was reported for 11.1% of the children, with a total of 10.6% of the children diagnosed with an inhalant allergy and 1.9% with a food allergy. Eczema was present in 20.0% of the children, and 8.3% of the children had asthma.

### 5.2. Diet Quality during Pregnancy

Associations of diet quality during pregnancy and allergic sensitization in children are presented in Table 2. In model 1, we observed a statistically significant association for inhalant allergic sensitization (OR = 1.06, 95% CI: 1.01, 1.12) (model 1, Table 2). However, this association was no longer statistically significant after adjustment for socioeconomic and lifestyle factors. In model 3, which was our main model, we observed no associations between diet quality during pregnancy and food allergic sensitization (OR = 1.04, 95% CI 0.92, 1.17) in children at the age of 10 years (model 3, Table 2). In line with our findings for allergic sensitization, we observed no statistically significant associations with self-reported physician-diagnosed inhalant (OR = 0.94, 95% CI: 0.88, 1.00) or food allergies (OR = 1.11, 95% CI: 0.91, 1.35), eczema (OR = 0.99, 95% CI: 0.93, 1.06), or asthma (OR = 0.93, 95% CI: 0.85, 1.03) in 10-year-old children (model 3, Table 2).

### 5.3. Diet Quality in Infancy

Associations of diet quality in infancy and allergic sensitization and atopic diseases in children are presented in Table 3. For diet quality in infancy, similar null findings were observed for inhalant allergic sensitization (OR = 0.99, 95% CI: 0.92, 1.06) and for food allergic sensitization (OR = 0.98, 95% CI: 0.86, 1.12) in model 3). Also, no associations were observed with inhalant (OR = 0.96, 95% CI: 0.87, 1.05) or food allergies (OR = 0.84, 95% CI: 0.70, 1.05) or with eczema or asthma in children around the age of 10 years (Table 3).

### 5.4. Diet Quality in Childhood

Table 4 presents associations of diet quality in childhood with the allergic outcomes. We observed no associations of diet quality in childhood with inhalant allergic sensitization (OR = 1.03, 95% CI: 0.96, 1.11) or food allergic sensitization (OR = 1.00, 95% CI: 0.88, 1.15) in childhood (model 3, Table 4). Similar null findings were observed for inhalant allergy (OR=1.05, 95% CI: 0.95, 1.15), food allergy (OR = 0.86, 95% CI: 0.69, 1.05), eczema (OR = 1.02, 95% CI: 0.95, 1.10), and asthma (OR = 1.03, 95% CI: 0.93, 1.15) (Table 4).

### 5.5. Additional Analyses

Associations were not statistically significantly different between Dutch and non-Dutch children for any of the outcomes (*p*-for-interaction >0.1). In line with this, sensitivity analyses restricted to children with a Dutch ethnic background only resulted in similar effect estimates as observed in the whole population (Supplemental Tables S4–S6). An exception was that among children with a Dutch ethnic background only, a higher diet quality during pregnancy was associated with a higher likelihood of inhalant allergic sensitization. However, we interpret this as a chance finding, since there was no association with any of the other outcomes; the interaction with ethnicity was not statistically significant; and because this finding would not remain if we would take into account multiple testing (*p* = 0.02). Also, analyses excluding children with any allergic disease in the first year of life resulted in similar findings (Supplemental Tables S7 and S8), except for an inverse association of diet quality in infancy with food allergy (OR = 0.64, 95% CI: 0.43, 0.95, *p* = 0.03), but not with any of the other outcomes. Analyses with a combination of allergic sensitization and allergic symptoms as the outcome (*n* = 642) versus no sensitization and symptoms (*n* = 1699) resulted in similar null findings (Supplemental Table S9). Additional adjustment for the other outcome variables did not affect the results (Supplemental Tables S10–S12). Also, excluding one component from the diet scores at a time, or additional adjustment for introduction of solid foods and day care attendance in the first year of life did not affect the results. We observed a significant interaction (*p* = 0.03) of infant diet quality with sex on food sensitization, and of infant diet quality with breastfeeding on eczema (*p* = 0.03), but not on any of the other outcomes (*p*-for-interaction ranging from 0.1 to 0.9). For none of the associations, we observed a significant interaction for maternal history of atopic diseases or child’s ethnic background (*p*-for-interaction >0.1). Stratification by sex suggested effect estimates in different directions (boys: OR = 0.88, 95% CI: 0.73, 1.06, model 3, girls: OR = 1.10, 95% CI: 0.91, 1.32, model 3), but none statistically significant. Similarly, after stratification by breastfeeding, no significant associations were observed in the different groups.

## 6. Discussion and Conclusions

In this large population-based study, we aimed to examine the associations between diet quality during pregnancy, infancy, and childhood with allergic sensitization, physician-diagnosed inhalant and food allergy, eczema, and asthma in mid-childhood. Overall, we observed no associations of overall diet quality during either pregnancy, infancy, or childhood with allergic sensitization or atopic diseases in children around the age of 10 years.

### 6.1. Interpretation and Comparison with Previous Studies

Although we observed a few associations of diet quality with atopic outcomes in our sensitivity analyses, these were not consistent and do not remain if multiple testing would be taken into account. In addition, the effect estimates were similar as observed in the main analyses. Our finding of a higher diet quality in infancy with lower odds of food allergy in additional analyses warrants caution and needs further study, as the prevalence of food allergy in these analyses is low (*n* = 18).

Previous studies mainly reported on associations of one particular time point in childhood (e.g., either pregnancy, infancy, or childhood) with different atopic outcomes. In this study, we examined diet at three different time points in early life. Although some previous studies observed an inverse association of overall diet during either pregnancy or infancy with atopic outcomes in childhood [21,25], we did not observe such associations. This is in line with a previous study in the United Kingdom that also observed no associations of data-driven dietary patterns in pregnancy with asthma or atopy in children around the age of seven years [22]. A recent systematic review suggested that a Mediterranean diet during pregnancy may only have an inverse association with asthma in the offspring in their first year of life, but not afterwards [19]. The longer time window between exposure and outcomes, and the measurement of atopic outcomes at the age of 10 years may therefore explain the absence of an association in our study, as children may outgrow some atopic diseases as they become older [37] and diet may have no long-term effects. Indeed, previous analyses in our cohort showed a positive association of adherence to a ‘Western-like’ dietary pattern in early life with asthma-related symptoms such as wheezing at the ages of three and four years [26], whereas we did not observe associations of diet quality with asthma at the age of 10 years in the current study. This suggests that any potential association between early-life diet and atopic outcomes may take place within a short term and may not persist into later childhood.

However, studies with shorter time windows between exposure and outcome also report inconsistent findings. Several cross-sectional studies examined associations of a predefined Mediterranean dietary pattern in childhood with allergic outcomes [18,19]. Inverse associations were reported in some of these studies, for example for diet in children aged six to seven years [38] and 10 to 12 years [39]. A study in the United Kingdom observed a positive association of a Western dietary pattern, which was high in processed foods, at the age of eight years with asthma, but no associations with allergic sensitization at the ages of eight and 11 years [27]. Finally, another study observed no associations of children’s adherence to a Mediterranean diet at the age of 6.5 years with atopy at the same age [21].

In addition to the time window of measurements, geographic area and cultural differences may play a role in the inconsistent associations observed in the different studies. A meta-analysis suggested, for example, that a Mediterranean diet may only be protective for atopic symptoms among children in the Mediterranean area [18]. A possible explanation for this may be the use of different Mediterranean diet indices, which may reflect slightly different food products. Also, a recent study in Singapore reported that a dietary pattern which was particularly high in fish and other seafood such as shellfish, at the age of one year was associated with less allergic sensitization to house dust mite at the ages of 18 months, but also at the age of five years [25]. The high fish intake may have driven the observed association. However, children and adults in the Netherlands have a relatively low fish and shellfish intake [40] and low variability. Therefore, these food groups do not drive large variations in our diet quality scores [30]. In addition, our diet quality scores were based on dietary guidelines, whereas most previous studies examined indices based on a traditional Mediterranean diet or data-driven dietary patterns and did not take into account dietary guidelines. However, these dietary patterns are population-specific and may not represent actual healthy diets. The absence of an association in our study may suggest that specific foods or nutrients, such as fish or fatty acids, rather than overall dietary patterns may be more relevant for the prevention of atopic outcomes in children. Further studies in specific populations are needed to confirm this.

### 6.2. Strengths and Limitations

The strengths of this study are its population-based, prospective design, the inclusion of a large number of participants, and the availability of numerous covariates. Also, we had detailed information on allergic sensitization to several common allergens relevant for school-age children, measured with skin prick tests. For these tests, we used the scanned area method to determine the wheal area, which is considered to be more accurate than measuring the average wheal diameter, and is recommended for use in academic research [34]. Furthermore, we analyzed overall dietary patterns, and not just single nutrients, which takes into account the interactions between different nutrients [17], and we had dietary data available at several time points throughout early life.

Several limitations of this study should also be considered. First, dietary intake during pregnancy, infancy, and childhood were assessed with FFQs, which are prone to measurement errors [41]. However, FFQs are commonly used in large epidemiological studies and have been shown to rank participants accurately according to their dietary intake [41]. In addition, in our study, we used validated, extensive, population-specific FFQs [30,31,33]. Although allergic sensitization was measured objectively using skin-prick tests, our other atopic outcomes were assessed with questionnaires filled out by the parents, which may have resulted in some misclassification. These questionnaires included questions on physician-diagnosed inhalant or food allergies, eczema, and asthma by any physician, but with no further details. However, we expect any misclassification to be unrelated to the exposure and therefore only resulting in random information bias. Furthermore, results for the associations of diet quality with objectively assessed allergic sensitization with skin prick tests and the self-reported atopic diseases were consistent. Despite that we were able to adjust the analyses for several confounders, some may not have been measured perfectly and there may be other possible confounding factors that we did not have available. Finally, most of the participants included in our study had a Dutch ethnic background, were, on average, highly educated, and had a high household income, which may limit the generalizability of our findings to other populations. However, in our sensitivity analyses restricted to participants with a Dutch ethnic background only similar results were obtained, suggesting no large bias due to ethnic background.

In conclusion, our findings suggest that overall diet quality in early life, either during pregnancy, infancy, or childhood, is not associated with the risk of allergic sensitization or atopic diseases in later childhood. Specific nutrients rather than overall dietary patterns may be more relevant for atopic outcomes in children and require further study.

## Figures and Tables

**Table 1:** Characteristics of the study population (*n* = 5225).

	*N* (%), Median (IQR), or Mean (SD)
**Maternal characteristics**	
Age at enrollment, years	31.7 (28.4–34.4)
Total energy intake, kcal/d (*n* = 4069)	2047 (1670–2439)
Diet quality score (*n* = 4069)	7.7 (1.6)
Educational level, higher	2751 (52.7%)
Household income, ≥2200 Euros per month	3234 (61.2%)
Parity, nulliparous	3027 (57.9%)
Prenatal pet exposure, yes	1800 (34.4)
Alcohol intake during pregnancy	
Never	2040 (39.0%)
Until pregnancy was known	722 (13.8%)
Occasionally during pregnancy	1949 (37.3%)
Frequently during pregnancy	514 (9.8%)
Smoking during pregnancy	
Never	3978 (76.1%)
Until pregnancy was known	484 (9.3%)
Continued during pregnancy	763 (14.6%)
Folic acid supplement use	
No	1084 (20.8%)
Started in the first 10 weeks of pregnancy	1745 (33.4%)
Started periconceptional	2395 (45.8%)
History of atopic disease, yes	2116(40.5%)
**Infant characteristics **	
Sex, female	2652 (50.8%)
Ethnic background, Dutch	3331 (63.7%)
Age at dietary assessment, months (*n* = 2796)	12.9 (12.7–13.9)
Total energy intake, kcal/d (*n* = 2796)	1261 (1058–1505)
Diet quality score (*n* = 2796)	4.3 (1.4)
Breastfeeding	
Never	508 (9.7%)
Four months partially	3401 (65.1%)
Four months exclusively	1315 (25.2%)
**Child characteristics **	
Age at dietary assessment, years	8.1 (8.0–8.2)
Total energy intake, kcal/d (*n* = 4066)	1461 (1240–1702)
Diet quality score (*n* = 4066)	4.5 (1.2)
Age at outcome assessment, years	9.7 (9.6–9.9)
Any allergic sensitization (*n* = 3911)	1357 (26.0%)
Inhalant allergic sensitization	1335 (25.6%)
Food allergic sensitization	298 (5.7%)
Any allergy (*n* = 4577)	579 (11.1%)
Inhalant allergy	554 (10.6%)
Food allergy	97 (1.9%)
Ever eczema (*n* = 4598)	1046 (20.0%)
Ever asthma (*n* = 4616)	432 (8.3%)

Values are means (±standard deviation (SD)) for continuous variables with a normal distribution, or medians (interquartile range (IQR)) for continuous variables with a skewed distribution, and absolute numbers (percentages) for categorical variables and are based on imputed data. Missing values for educational level (5.0%), household income (18.9%), parity (2.8%), prenatal pet exposure (20.6%), alcohol intake (during pregnancy (14.2%), smoking during pregnancy (17.1%), folic acid supplement use (28.2%), history of atopic disease (17.2%), child ethnic background (0.2%), and breastfeeding (30.1%) were multiple imputed (*n* = 10 imputations).

**Table 2:** Associations of diet quality in pregnancy with allergic sensitization and allergic diseases in childhood at the age of 10 years.

	OR (95% CI) Per 1 Point Higher Diet Quality Score		
	Model 1	Model 2	Model 3
Any allergic sensitization (*n* = 1019/2960)	1.06 (1.00, 1.11)	1.06 (0.99, 1.13)	1.05 (0.99, 1.13)
Inhalant allergic sensitization (*n* = 1002/2960)	**1.06 (1.01, 1.12)**	1.06 (0.99, 1.13)	1.06 (0.99, 1.13)
Food allergic sensitization (*n* = 224/2960)	1.04 (0.94, 1.14)	1.04 (0.92, 1.16)	1.04 (0.92, 1.17)
Any allergy (*n* = 449/3588)	0.96 (0.90, 1.03)	0.96 (0.88, 1.04)	0.96 (0.88, 1.04)
Inhalant allergy (*n* = 427/3588)	0.95 (0.89, 1.00)	0.94 (0.88, 1.00)	0.94 (0.88, 1.00)
Food allergy (*n* = 69/3588)	1.06 (0.91, 1.24)	1.04 (0.86, 1.25)	1.11 (0.91, 1.35)
Ever eczema (*n* = 840/3600)	1.03 (0.98, 1.08)	1.00 (0.94, 1.06)	0.99 (0.93, 1.06)
Ever asthma (*n* = 319/3610)	**0.91 (0.84, 0.98)**	0.94 (0.86, 1.03)	0.93 (0.85, 1.03)

Values are odds ratios with 95% confidence intervals (CIs) from logistic regression analyses, for allergic sensitization or atopic disease per 1 point higher diet quality score. Numbers (*n*) represent cases/total population with valid data included in the analyses. Bold values represent *p*-value < 0.05. Model 1: Sex, ethnic background, age at outcome assessment, total energy intake. Model 2: Maternal BMI at enrollment, maternal educational level, household income, parity, prenatal pet exposure, alcohol intake during pregnancy, smoking during pregnancy, folic acid supplements during pregnancy, maternal history of atopic disease, breastfeeding. Model 3: Diet quality in infancy and childhood.

**Table 3:** Associations of diet quality in infancy with allergic sensitization and allergic diseases in childhood at the age of 10 years.

	OR (95% CI) Per 1 Point Higher Diet Quality Score		
	Model 1	Model 2	Model 3
Any allergic sensitization (*n* = 823/2456)	1.00 (0.94, 1.06)	1.00 (0.94, 1.07)	0.99 (0.92, 1.06)
Inhalant allergic sensitization (*n* = 808/2456)	1.00 (0.94, 1.07)	1.00 (0.94, 1.07)	0.99 (0.92, 1.06)
Food allergic sensitization (*n* = 173/2456)	0.99 (0.93, 1.05)	0.99 (0.87, 1.12)	0.98 (0.86, 1.12)
Any allergy (*n* = 316/2519)	0.94 (0.87, 1.02)	0.94 (0.86, 1.03)	0.94 (0.86, 1.04)
Inhalant allergy (*n *= 302/2519)	0.95 (0.87, 1.04)	0.96 (0.87, 1.05)	0.96 (0.87, 1.05)
Food allergy (*n* = 58/2519)	0.84 (0.68, 1.03)	0.82 (0.67, 1.01)	0.84 (0.70, 1.05)
Ever eczema (*n* = 586/2543)	1.00 (0.97, 1.04)	1.00 (0.93, 1.07)	1.00 (0.93, 1.08)
Ever asthma (*n* = 236/2542)	0.95 (0.86, 1.05)	0.96 (0.86, 1.06)	0.96 (0.86, 1.07)

Values are odds ratios with 95% confidence intervals (CIs) from logistic regression analyses, for allergic sensitization or atopic disease per 1 point higher diet quality score. Numbers (*n*) represent cases/total population with valid data included in the analyses. Model 1: Sex, ethnic background, age at outcome assessment, total energy intake. Model 2: Maternal BMI at enrollment, maternal educational level, household income, parity, prenatal pet exposure, alcohol intake during pregnancy, smoking during pregnancy, folic acid supplements during pregnancy, maternal history of atopic disease, breastfeeding. Model 3: Diet quality in pregnancy and childhood.

**Table 4:** Associations of diet quality in childhood with allergic sensitization and allergic diseases in childhood at the age of 10 years.

	OR (95% CI) Per 1 Point Higher Diet Quality Score		
	Model 1	Model 2	Model 3
Any allergic sensitization (*n* = 1012/3017)	1.03 (0.97, 1.10)	1.04 (0.97, 1.11)	1.03 (0.96, 1.11)
Inhalant allergic sensitization (*n* = 994/3017)	1.04 (0.97, 1.11)	1.04 (0.97, 1.12)	1.03 (0.96, 1.11)
Food allergic sensitization (*n* = 218/3017)	0.99 (0.93, 1.06)	0.99 (0.87, 1.13)	1.00 (0.88, 1.15)
Any allergy (*n* = 463/3750)	1.00 (0.92, 1.09)	1.02 (0.94, 1.12)	1.04 (0.95, 1.14)
Inhalant allergy (*n* = 445/3750)	1.00 (0.92, 1.09)	1.03 (0.94, 1.12)	1.05 (0.95, 1.15)
Food allergy (*n* = 79/3750)	0.88 (0.72, 1.06)	0.85 (0.69, 1.04)	0.86 (0.69, 1.05)
Ever eczema (*n* = 850/3766)	1.02 (0.98, 1.05)	1.01 (0.95, 1.09)	1.02 (0.95, 1.10)
Ever asthma (*n* = 335/3776)	0.97 (0.92, 1.02)	1.01 (0.91, 1.12)	1.03 (0.93, 1.15)

Values are odds ratios with 95% confidence intervals (CIs) from logistic regression analyses, for allergic sensitization or atopic disease per 1 point higher diet quality score. Numbers (*n*) represent cases/total population with valid data included in the analyses. Model 1: Sex, ethnic background, age at outcome assessment, total energy intake. Model 2: Maternal BMI at enrollment, maternal educational level, household income, parity, prenatal pet exposure, alcohol intake during pregnancy, smoking during pregnancy, folic acid supplements during pregnancy, maternal history of atopic disease, breastfeeding. Model 3: Diet quality in pregnancy and infancy.

## Supplementary Materials

The following are available online at www.mdpi.com/2072-6643/9/8/841/s1, Figure S1: Flowchart of the population for analyses on diet quality during pregnancy, Figure S2: Flowchart of the population for analyses on infant diet quality, Figure S3: Flowchart of the population for analyses on childhood diet quality, Table S1: Components and cut-offs included in the diet quality score for pregnant women, Table S2: Components and cut-offs included in the diet quality score for 1-year-old children, Table S3: Components and cut-offs included in the diet quality score for 8-year-old children, Table S4: Associations of diet quality in pregnancy with allergic sensitization and allergic diseases in 10-year-old children with a Dutch ethnic background, Table S5. Associations of diet quality in infancy with allergic sensitization and allergic diseases in 10-year-old children with a Dutch ethnic background, Table S6. Associations of diet quality in childhood with allergic sensitization and allergic diseases in 10-year-old children with a Dutch ethnic background, Table S7. Associations of diet quality in infancy with allergic sensitization and allergic diseases in 10-year-old children without an allergic disease in the first year of life, Table S8. Associations of diet quality in childhood with allergic sensitization and allergic diseases in 10-year-old children without an allergic disease in the first year of life, Table S9. Associations of diet quality with a combination of allergic sensitization and any allergic symptom in 10-year-old children, Table S10. Associations of diet quality in pregnancy with allergic sensitization and allergic diseases in 10-year-old children, with additional adjustment for the other outcomes variables, Table S11. Associations of diet quality in infancy with allergic sensitization and allergic diseases in 10-year-old children, with additional adjustment for the other outcomes variables, Table S12. Associations of diet quality in childhood with allergic sensitization and allergic diseases in 10-year-old children, with additional adjustment for the other outcomes variables.
