# Supplementary material for: Diet Quality throughout Early Life in Relation to Allergic Sensitization and Atopic Diseases in Childhood
Source: Nutrients. 2017 Aug 5;9(8):841. doi: 10.3390/nu9080841 (PMC5579634; doi:10.3390/nu9080841)
Supplement: Supplementary file 1 [file nutrients-09-00841-s001.pdf]

nutrients

Supplemental materials

Figure S1. Flowchart of the population for analyses on diet quality during pregnancy

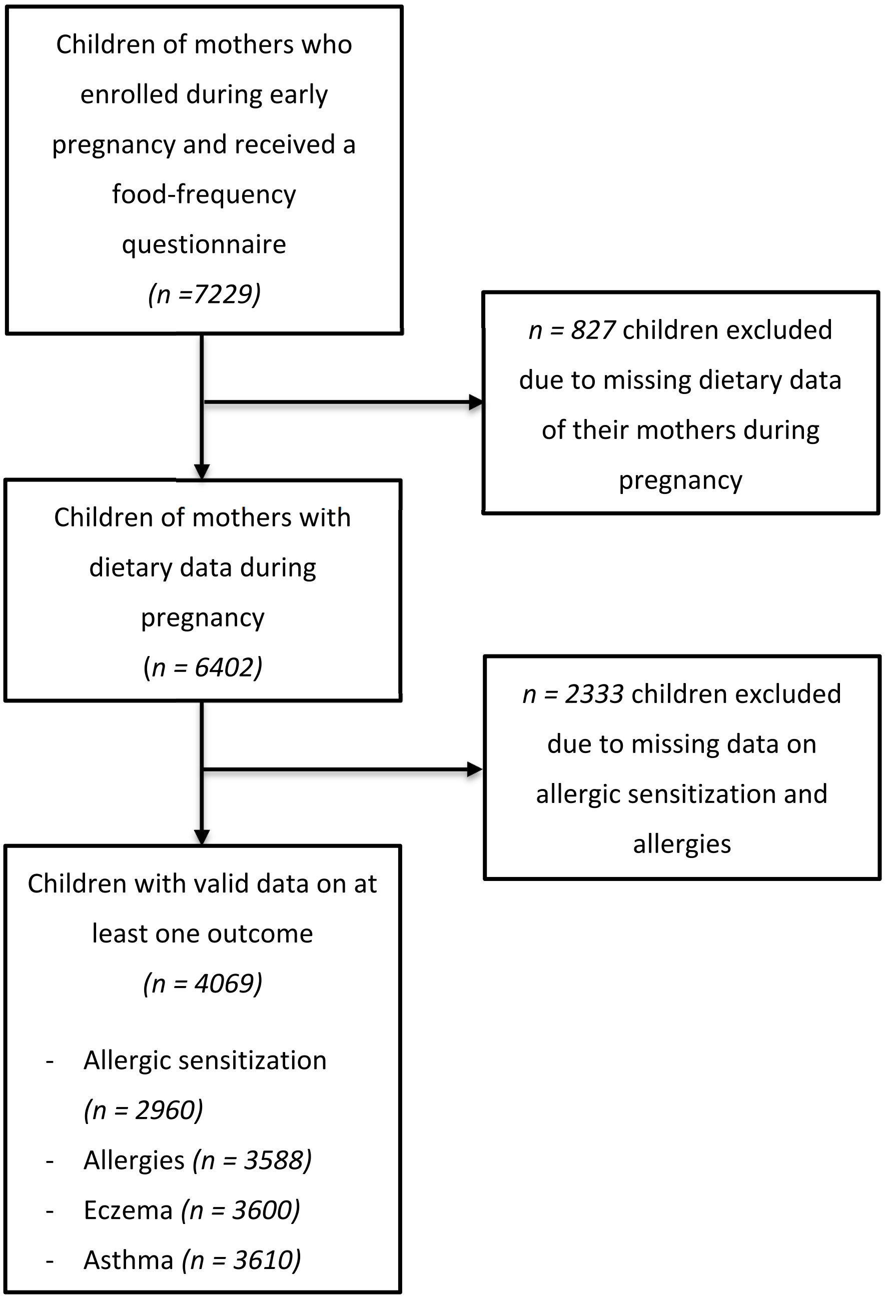

Figure S2. Flowchart of the population for analyses on infant diet quality

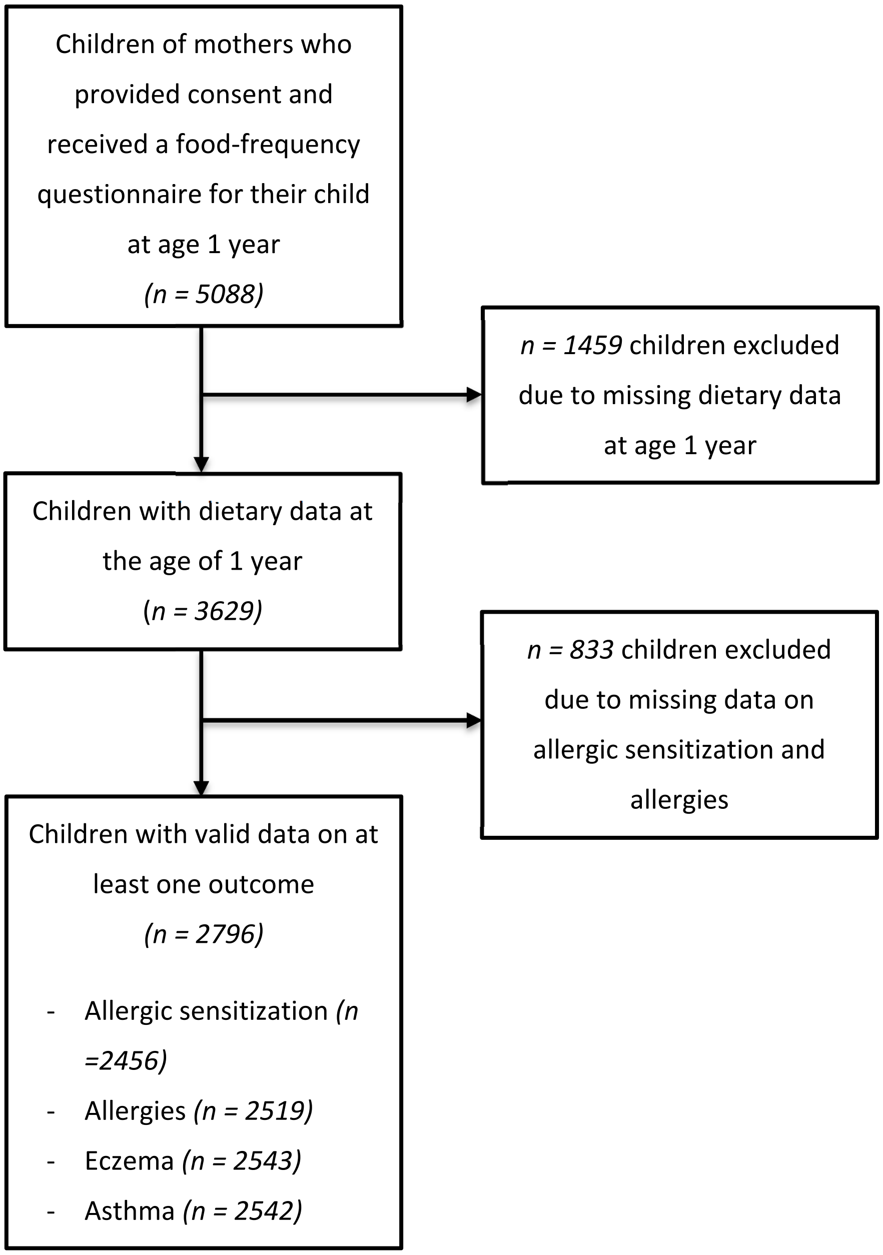

Figure S3. Flowchart of the population for analyses on childhood diet quality

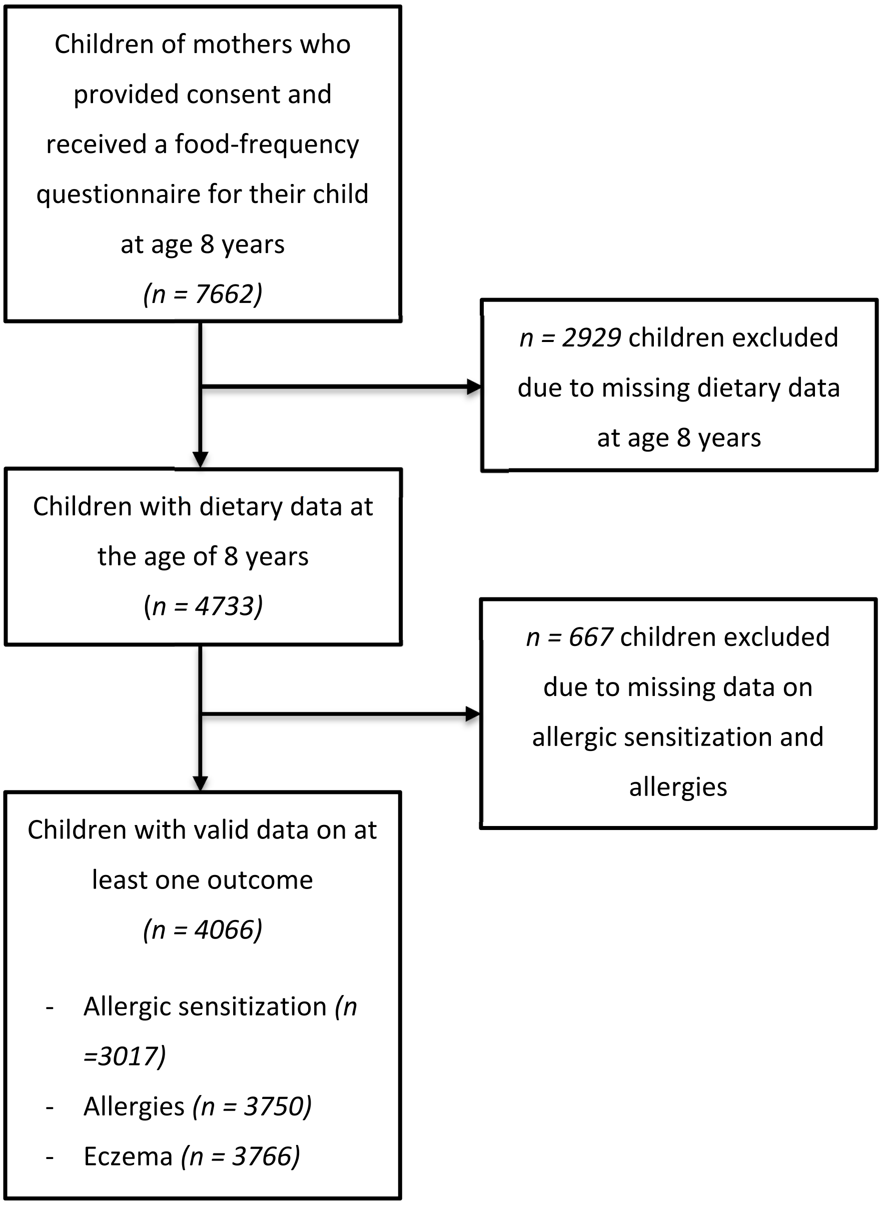

Table S1. Components and cut-offs included in the diet quality score for pregnant women.

Component	Cut-off value
Vegetables	≥ 200 g/day
Fruit	≥ 200 g/day
Whole-grains	≥ 90 g/day
Legumes	≥ 135 g/week
Nuts	≥ 15 g/day
Dairy	≥ 300 g/day
Fish	≥ 100 g/week
Tea	≥ 30 g/day
Red meat	≤ 375 g/week
Sugar-containing beverages	≤ 150 g/day
Salt	≤ 6 g/day
Grain quality	Ratio whole grains of total grains
Soft fats and oils	Ratio soft fats and oils of total fat
Alcohol	Yes/no
Folic supplement use	Periconceptional /first ten weeks/not

Table S2. Components and cut-offs included in the diet quality score for 1-year-old children.

Component	Cut-off value
Vegetables	≥ 100 g/day
Fruit	≥ 150 g/day
Bread and cereals	≥ 70 g/day
Rice, pasta, potatoes, and legumes	≥ 70 g/day
Dairy	≥ 350 g/day
Meat, eggs, and meat substitutes	≥ 35 g/day
Fish	≥ 15 g/day
Oils and fats	≥ 25 g/day
Candy and snacks	≤ 20 g/day
Sugar-sweetened beverages	≤ 100 g/day

Table S3. Components and cut-offs included in thediet quality score for 8-year-old children

Component	Cut-off value
Vegetables	≥150 g/day
Fruit	≥150 g/day
Whole-grains	≥90 g/day
Fish	≥ 60 g/week
Legumes	≥ 84 g/week
Nuts	≥ 15 g/day
Dairy	≥ 300 g/day
Oils and soft or liquid fats	≥ 30 g/day
Sugar-containing beverages	≤ 150 g/day
High-fat and processed meat	≤ 250 g/week

Table S4. Associations of diet quality in pregnancy with allergic sensitization and allergic diseases in 10-year-old children with a Dutch ethnic background

	**OR (95%CI) per 1 point higher diet quality score**		
	Model 1	Model 2	Model 3
Any allergic sensitization (*n* = 597/1915)	1.09 (1.02, 1.17)	1.10 (1.01, 1.20)	1.11 (1.01, 1.21)
Inhalant allergic sensitization (*n* = 588/1915)	1.09 (1.02, 1.17)	1.11 (1.02, 1.21)	1.12 (1.02, 1.23)
Food allergic sensitization (*n* = 130/1915)	1.05 (0.92, 1.19)	1.05 (0.89, 1.23)	1.06 (0.90, 1.25)
Any allergy (*n* = 278/2487)	1.01 (0.92, 1.10)	0.98 (0.88, 1.08)	0.98 (0.88, 1.10)
Inhalant allergy (*n* = 264/2487)	0.98 (0.91, 1.06)	0.97 (0.90, 1.05)	0.97 (0.90, 1.05)
Food allergy (*n* = 42/2487)	1.14 (0.94, 1.38)	1.01 (0.80, 1.30)	1.08 (0.84, 1.40)
Eczema (*n* = 577/2518)	1.05 (0.99, 1.12)	1.02 (0.94, 1.10)	1.01 (0.93, 1.10)
Asthma (*n* = 195/2508)	0.96 (0.87, 1.07)	0.99 (0.88, 1.12)	0.97 (0.86, 1.11)

Values are odds ratios with 95% confidence intervals (CIs) from logistic regression analyses, for allergic sensitization or atopic disease per 1 point higher diet quality score. Numbers (n) represent cases/total population with valid data included in the analyses.
Model 1: Sex, age at outcome assessment, total energy intake.
Model 2: Maternal BMI at enrollment, maternal educational level, household income, parity, prenatal pet exposure, alcohol intake during pregnancy, smoking during pregnancy, folic acid supplements during pregnancy, maternal history of atopic disease, breastfeeding.
Model 3: Diet quality in infancy and childhood.

Table S5. Associations of diet quality in infancy with allergic sensitization and allergic diseases in 10-year-old children with a Dutch ethnic background

	**OR (95%CI) per 1 point higher diet quality score**		
	Model 1	Model 2	Model 3
Any allergic sensitization (*n* = 533/1720)	1.00 (0.93, 1.09)	1.00 (0.92, 1.08)	0.98 (0.90, 1.06)
Inhalant allergic sensitization (*n* = 524/1720)	1.00 (0.93, 1.09)	1.00 (0.92, 1.09)	0.98 (0.90, 1.07)
Food allergic sensitization (*n* = 107/1720)	0.96 (0.83, 1.11)	0.94 (0.79, 1.10)	0.93 (0.78, 1.10)
Any allergy (*n* = 214/1835)	0.93 (0.83, 1.04)	0.92 (0.82, 1.04)	0.92 (0.82, 1.04)
Inhalant allergy (*n* = 205/1835)	0.94 (0.84, 1.05)	0.94 (0.83, 1.05)	0.94 (0.83, 1.05)
Food allergy (*n* = 36/1835)	0.85 (0.65, 1.10)	0.82 (0.62, 1.07)	0.84 (0.64, 1.11)
Eczema (*n* = 417/1854)	1.02 (0.94, 1.11)	1.01 (0.93, 1.10)	1.01 (0.93, 1.11)
Asthma (*n* = 158/1849)	0.98 (0.86, 1.11)	0.98 (0.86, 1.11)	0.98 (0.85, 1.12)

Values are odds ratios with 95% confidence intervals (CIs) from logistic regression analyses, for allergic sensitization or atopic disease per 1 point higher diet quality score. Numbers (n) represent

cases/total population with valid data included in the analyses.
Model 1: Sex, age at outcome assessment, total energy intake.
Model 2: Maternal BMI at enrollment, maternal educational level, household income, parity, prenatal pet exposure, alcohol intake during pregnancy, smoking during pregnancy, folic acid supplements during pregnancy, maternal history of atopic disease, breastfeeding.
Model 3: Diet quality in pregnancy and childhood.

Table S6. Associations of diet quality in childhood with allergic sensitization and allergic diseases in 10-year-old children with a Dutch ethnic background

	**OR (95%CI) per 1 point higher diet quality score**		
	Model 1	Model 2	Model 3
Any allergic sensitization (*n* = 613/1997)	1.03 (0.95, 1.12)	1.04 (0.95, 1.14)	1.02 (0.93, 1.12)
Inhalant allergic sensitization (*n* = 604/1997)	1.04 (0.95, 1.13)	1.05 (0.96, 1.14)	(0.94, 1.13)
Food allergic sensitization (*n* = 127/1997)	0.96 (0.82, 1.11)	0.96 (0.81, 1.14)	0.97 (0.82, 1.16)
Any allergy (*n* = 298/2616)	0.95 (0.86, 1.06)	0.97 (0.86, 1.08)	0.98 (0.87, 1.11)
Inhalant allergy (*n* = 285/2616)	0.95 (0.85, 1.06)	0.97 (0.87, 1.09)	0.99 (0.87, 1.11)
Food allergy (*n* = 48/2616)	0.83 (0.65, 1.06)	0.77 (0.59, 1.01)	0.77 (0.58, 1.02)
Eczema (*n* = 589/2639)	1.01 (0.93, 1.09)	1.00 (0.92, 1.09)	1.01 (0.92, 1.10)
Asthma (*n* = 219/2632)	1.01 (0.89, 1.14)	1.05 (0.92, 1.19)	1.07 (0.94, 1.22)

Values are odds ratios with 95% confidence intervals (CIs) from logistic regression analyses, for allergic sensitization or atopic disease per 1 point higher diet quality score. Numbers (n) represent cases/total population with valid data included in the analyses.
Model 1: Sex, age at outcome assessment, total energy intake.
Model 2: Maternal BMI at enrollment, maternal educational level, household income, parity, prenatal pet exposure, alcohol intake during pregnancy, smoking during pregnancy, folic acid supplements during pregnancy, maternal history of atopic disease, breastfeeding.
Model 3: Diet quality in pregnancy and infancy.

Table S7. Associations of diet quality in infancy with allergic sensitization and allergic diseases in 10-year-old children without an allergic disease in the first year of life

	**OR (95%CI) per 1 point higher diet quality score**		
	Model 1	Model 2	Model 3
Any allergic sensitization (*n* =466/1680)	0.95 (0.88, 1.03)	0.96 (0.88, 1.04)	0.95 (0.87, 1.03)
Inhalant allergic sensitization (*n* =460/1680)	0.95 (0.88, 1.03)	0.96 (0.88, 1.04)	0.95 (0.87, 1.03)
Food allergic sensitization (*n* =70/1680)	0.92 (0.76, 1.11)	0.92 (0.76, 1.12)	0.91 (0.75, 1.11)
Any allergy (*n* =155/1749)	0.92 (0.82, 1.05)	0.93 (0.82, 1.06)	0.93 (0.81, 1.06)
Inhalant allergy (*n* =149/1749)	0.93 (0.82, 1.06)	0.95 (0.83, 1.08)	0.94 (0.82, 1.08)
Food allergy (*n* =18/1749)	0.67 (0.45, 0.98)	0.64 (0.43, 0.94)	0.64 (0.43, 0.95)
Eczema (*n* =265/1770)	1.00 (0.91, 1.10)	1.00 (0.90, 1.10)	0.99 (0.90, 1.10)
Asthma (*n* =110/1774)	0.94 (0.83, 1.07)	0.96 (0.83, 1.12)	0.96 (0.82, 1.12)

Values are odds ratios with 95% confidence intervals (CIs) from logistic regression analyses, for allergic sensitization or atopic disease per 1 point higher diet quality score. Numbers (n) represent cases/total population with valid data included in the analyses.
Model 1: Sex, ethnic background, age at outcome assessment, total energy intake.
Model 2: Maternal BMI at enrollment, maternal educational level, household income, parity, prenatal pet exposure, alcohol intake during pregnancy, smoking during pregnancy, folic acid supplements during pregnancy, maternal history of atopic disease, breastfeeding.
Model 3: Diet quality in pregnancy and childhood.

Table S8. Associations of diet quality in childhood with allergic sensitization and allergic diseases in 10-year-old children without an allergic disease in the first year of life

	**OR (95%CI) per 1 point higher diet quality score**		
	Model 1	Model 2	Model 3
Any allergic sensitization (*n* =474/1704)	1.00 (0.91, 1.09)	1.02 (0.92, 1.12)	1.01 (0.91, 1.12)
Inhalant allergic sensitization (*n* =468/1704)	1.00 (0.91, 1.09)	1.02 (0.92, 1.12)	1.01 (0.91, 1.12)
Food allergic sensitization (*n* =70/1704)	0.96 (0.78, 1.18)	0.99 (0.79, 1.23)	1.00 (0.79, 1.26)
Any allergy (*n* =174/2120)	1.01 (0.88, 1.16)	1.06 (0.92, 1.22)	1.08 (0.93, 1.25)
Inhalant allergy (*n* =168/2120)	1.01 (0.88, 1.16)	1.06 (0.92, 1.23)	1.08 (0.93, 1.26)
Food allergy (*n* =20/2120)	0.89 (0.61, 1.31)	0.86 (0.57, 1.27)	0.92 (0.60, 1.39)
Eczema (*n* =313/2136)	1.07 (0.97, 1.19)	1.09 (0.97, 1.21)	1.11 (0.99, 1.25)
Asthma (*n* =121/2139)	1.00 (0.85, 1.17)	1.04 (0.88, 1.23)	1.07 (0.91, 1.27)

Values are odds ratios with 95% confidence intervals (CIs) from logistic regression analyses, for allergic sensitization or atopic disease per 1 point higher diet quality score. Numbers (n) represent

cases/total population with valid data included in the analyses.
Model 1: Sex, ethnic background, age at outcome assessment, total energy intake.Model 2: Maternal BMI at enrollment, maternal educational level, household income, parity, prenatal pet exposure, alcohol intake during pregnancy, smoking during pregnancy, folic acid supplements during pregnancy, maternal history of atopic disease, breastfeeding.
Model 3: Diet quality in pregnancy and infancy.

Table S9. Associations of diet quality with a combination of allergic sensitization and any allergic symptom in 10-year-old children

	**OR (95%CI) per 1 point higher diet quality score**
Diet quality during pregnancy (489/1783)	
Model 1	1.04 (0.97, 1.12)
Model 2	1.04 (0.95, 1.13)
Model 3	1.03 (0.94, 1.13)
Diet quality in infancy (413/1573)	
Model 1	1.01 (0.95, 1.08)
Model 2	1.01 (0.92, 1.10)
Model 3	1.00 (0.92, 1,01)
Diet quality in childhood (510/1928)	
Model 1	1.05 (0.96, 1.15)
Model 2	1.07 (0.97, 1.17)
Model 3	1.08 (0.98, 1.19)

Values are odds ratios with 95% confidence intervals (CIs) from logistic regression analyses, for allergic sensitization and any allergic symptom per 1 point higher diet quality score. Numbers (n) represent cases/total population with valid data included in the analyses.
Model 1: Sex, ethnic background, age at outcome assessment, total energy intake.
Model 2: Maternal BMI at enrollment, maternal educational level, household income, parity, prenatal pet exposure, alcohol intake during pregnancy, smoking during pregnancy, folic acid supplements during pregnancy, maternal history of atopic disease, breastfeeding.
Model 3: Diet quality at the two other time points.

Table S10. Associations of diet quality in pregnancy with allergic sensitization and allergic diseases in 10-year-old children, with additional adjustment for the other outcomes variables

	**OR (95%CI) per 1 point higher diet quality score**		
	Model 1	Model 2	Model 3
Any allergic sensitization (*n* =1019/2960)	1.06 (1.00, 1.11)	1.06 (0.99, 1.13)	1.07 (1.00, 1.16)
Inhalant allergic sensitization (*n* =1002/2960)	1.06 (1.01, 1.12)	1.06 (0.99, 1.13)	1.08 (1.00, 1.16)
Food allergic sensitization (*n* =224/2960)	1.04 (0.94, 1.14)	1.04 (0.92, 1.16)	1.08 (0.94, 1.24)
Any allergy (*n* =449/3588)	0.96 (0.90, 1.03)	0.96 (0.88, 1.04)	0.94 (0.85, 1.04)
Inhalant allergy (*n* =427/3588)	0.95 (0.89, 1.00)	0.94 (0.88, 1.00)	0.93 (0.86, 1.00)
Food allergy (*n* =69/3588)	1.06 (0.91, 1.24)	1.04 (0.86, 1.25)	1.02 (0.83, 1.27)
Eczema (*n* =840/3600)	1.03 (0.98, 1.08)	1.00 (0.94, 1.06)	1.00 (0.94, 1.07)
Asthma (*n* =319/3610)	0.91 (0.84, 0.98)	0.94 (0.86, 1.03)	0.95 (0.86, 1.05)

Values are odds ratios with 95% confidence intervals (CIs) from logistic regression analyses, for allergic sensitization or atopic disease per 1 point higher diet quality score. Numbers (n) represent cases/total population with valid data included in the analyses.
Model 1: Sex, ethnic background, age at outcome assessment, total energy intake.
Model 2: Maternal BMI at enrollment, maternal educational level, household income, parity, prenatal pet exposure, alcohol intake during pregnancy, smoking during pregnancy, folic acid supplements during pregnancy, maternal history of atopic disease, breastfeeding.
Model 3: Other outcome variables.

Table S11. Associations of diet quality in infancy with allergic sensitization and allergic diseases in 10-year-old children, with additional adjustment for the other outcomes variables

	**OR (95%CI) per 1 point higher diet quality score**		
	Model 1	Model 2	Model 3
Any allergic sensitization (*n* =823/2456)	1.00 (0.94, 1.06)	1.00 (0.94, 1.07)	0.99 (0.92, 1.07)
Inhalant allergic sensitization (*n* =808/2456)	1.00 (0.94, 1.07)	1.00 (0.94, 1.07)	0.99 (0.92, 1.07)
Food allergic sensitization (*n* =173/2456)	0.99 (0.93, 1.05)	0.99 (0.87, 1.12)	1.03 (0.88, 1.19)
Any allergy (*n* =316/2519)	0.94 (0.86, 1.02)	0.94 (0.86, 1.03)	0.93 (0.83, 1.04)
Inhalant allergy (*n* =302/2519)	0.95 (0.88, 1.04)	0.96 (0.87, 1.05)	0.94 (0.84, 1.06)
Food allergy (*n* =58/2519)	0.84 (0.68, 1.03)	0.82 (0.67, 1.01)	0.79 (0.62, 1.02)
Eczema (*n* =586/2543)	1.00 (0.97, 1.04)	1.00 (0.93, 1.07)	1.01 (0.94, 1.09)
Asthma (*n* =236/2542)	0.95 (0.86, 1.05)	0.96 (0.86, 1.06)	0.98 (0.87, 1.09)

Values are odds ratios with 95% confidence intervals (CIs) from logistic regression analyses, for allergic sensitization or atopic disease per 1 point higher diet quality score. Numbers (n) represent

cases/total population with valid data included in the analyses.
Model 1: Sex, ethnic background, age at outcome assessment, total energy intake.
Model 2: Maternal BMI at enrollment, maternal educational level, household income, parity, prenatal pet exposure, alcohol intake during pregnancy, smoking during pregnancy, folic acid supplements during pregnancy, maternal history of atopic disease, breastfeeding.
Model 3: Other outcome variables.

Table S12. Associations of diet quality in childhood with allergic sensitization and allergic diseases in 10-year-old children, with additional adjustment for the other outcomes variables

	**OR (95%CI) per 1 point higher diet quality score**		
	Model 1	Model 2	Model 3
Any allergic sensitization (*n* =1012/3017)	1.03 (0.97, 1.10)	1.04 (0.97, 1.11)	1.02 (0.94, 1.11)
Inhalant allergic sensitization (*n* =994/3017)	1.04 (0.97, 1.11)	(0.97, 1.12)	1.03 (0.95, 1.11)
Food allergic sensitization (*n* =218/3017)	0.99 (0.93, 1.06)	0.99 (0.87, 1.13)	0.97 (0.82, 1.15)
Any allergy (*n* =463/3750)	1.00 (0.92, 1.09)	1.02 (0.94, 1.12)	1.01 (0.89, 1.15)
Inhalant allergy (*n* =445/3750)	1.00 (0.92, 1.08)	1.03 (0.94, 1.12)	1.02 (0.90, 1.16)
Food allergy (*n* =79/3750)	0.88 (0.72, 1.06)	0.85 (0.69, 1.04)	0.84 (0.66, 1.08)
Eczema (*n* =850/3766)	1.02 (0.98, 1.05)	1.01 (0.94, 1.09)	1.01 (0.93, 1.09)
Asthma (*n* =335/3776)	0.97 (0.92, 1.02)	1.01 (0.91, 1.12)	0.99 (0.89, 1.11)

Values are odds ratios with 95% confidence intervals (CIs) from logistic regression analyses, for allergic sensitization or atopic disease per 1 point higher diet quality score. Numbers (n) represent cases/total population with valid data included in the analyses.
Model 1: Sex, ethnic background, age at outcome assessment, total energy intake.
Model 2: Maternal BMI at enrollment, maternal educational level, household income, parity, prenatal pet exposure, alcohol intake during pregnancy, smoking during pregnancy, folic acid supplements during pregnancy, maternal history of atopic disease, breastfeeding.
Model 3: Other outcome variables.
